# Community challenges when using large plastic bottles for Solar Energy Disinfection of Water (SODIS)

**DOI:** 10.1186/s12889-016-3535-6

**Published:** 2016-09-05

**Authors:** Preeti Borde, Khalifa Elmusharaf, Kevin G. McGuigan, Michael B. Keogh

**Affiliations:** 1 Department of Basic Biomedical Sciences, RCSI, Manama, Bahrain; 2 Graduate Entry Medical School, University of Limerick, Limerick, Ireland; 3 Royal College of Surgeons in Ireland, Dublin 2, Ireland

**Keywords:** SODIS, Scale-up, Community implementation

## Abstract

**Background:**

Communities living in developing countries as well as populations affected by natural or man-made disasters can be left at great risk from water related diseases, especially those spread through the faecal-oral route. Conventional water treatments such as boiling and chlorination can be effective but may prove costly for impoverished communities. Solar water disinfection (SODIS) has been shown to be a cheap and effective way for communities to treat their water. The exposure to sunlight is typically carried out in small volume plastic beverage bottles (up to 2 l). Given the water requirements of consumption and basic personal hygiene, this may not always meet the needs of communities. Recent work has shown 19-L plastic water dispenser containers to be effective SODIS reactors, comparable in efficacy to PET bottles. In this paper we outline the need for studying SODIS in large volumes and discuss 4 main associated challenges.

**Discussion:**

Apart from clean water needed for consumption, access to adequate water is essential for sanitation and hygiene. Contamination of treated water through unwashed hands or vessels contributes heavily to the spread of water borne pathogens in communities. Traditional water treatments such as boiling and chlorination can be effective but may prove financially burdensome for low income communities. SODIS in large vessels could be used as a simple method to meet water requirements in low income and disaster affected populations. However, there have been some concerns associated with the conventional SODIS method; we identify the main ones to be: (1) cold or cloudy weather; (2) the fear of leaching in plastic bottles; (3) water turbidity, and; (4) community acceptance.

**Summary:**

The application of SODIS in large bottles like WDCs has the potential to be an efficient and cost effective method of disinfecting water, either for consumption until more rigorous water treatments can be put in place, or for sanitation and hygiene to curb the spread of fecal contamination. Further research is needed that can address some of the limitations and challenges associated with the use of large bottles for SODIS.

## Background

The World Health Organization (WHO) estimates that around 10 % of the global burden of disease can be attributed to lack of adequate drinking water, poor hygiene, and inefficient sanitation [1]. Unsafe water is the main reason for diarrheal diseases such as dysentery and cholera, preventable and treatable disorders which claim the lives of around 1.5 million people every year, around 760,000 of these being children under 5 years [2]. Water treatment techniques such as filtration, boiling, chlorination and ultra violet radiation, while effective may be too costly to use in developing countries or emergency situations. Solar water disinfection (SODIS) is a simple and cheap method which uses natural sunlight to treat contaminated water filled into transparent plastic containers and exposed to direct sunlight for up to 6 h. SODIS has conventionally been implemented using 1-2 L polyethylene terephthalate (PET) bottles which have shown good characteristics of microbiological inactivation in natural sunlight [3–6]. To date, most SODIS based research has focused on using these 2 L PET bottles [5, 7, 8].

The aim of this debate article is to identify the needs for SODIS in large volume bottles as well as to debate their limitations. The findings of this article will help to inform the global conversations around access to clean and safe water in resource-limited settings and to help develop appropriate and sustainable strategies at community level to enhance compliance with SODIS.

## Discussion

### What is the Need for SODIS in Large Volumes?

SODIS success stories are described from many groups under various field conditions, all of which strengthen solar water disinfection’s position as a sustainable, cost effective method of acquiring clean drinking water in developing countries [9–11]. The impact on health of consumers of SODIS treated water has been closely followed in several countries such as Kenya, India and Cameroon and the data is encouraging with decreases in prevalence of diarrhea and other water borne diseases being reported among the intervention groups [12–14].

Unfortunately, the capacities of commercial PET bottles, which usually come in sizes that can hold up to 2 L of water, make them less suitable for disinfecting large quantities of water unless a number are put to use at the same time. This limits its sustained everyday use as a water treatment method owing mainly to the time and effort associated with setting up and keeping track of multiple SODIS bottles. This is an unfortunate restriction as SODIS holds several advantages over other treatment interventions such as boiling and chlorination in that apart from being low cost and easy to implement, it also uses up a minimal amount of consumables [3].

According to humanitarian agencies, it has been estimated that water requirements for consumption and hygiene based on basic survival needs are 7.5–15 l per person per day dependent on climate, sanitation facilities, social and cultural norms etc. [15]. We recently showed 19 l polycarbonate (PC) Water dispenser containers (WDCs) were as effective at SODIS treatment for *E. coli* or *E. faecalis* as 2 L PET bottles [16]. These larger water containers could be valuable in emergency settings as well as to impoverished communities.

#### Community needs

A large body of research has highlighted the strong link between improvements in sanitation, simple personal hygiene and interventions to improve water quality at point of use with a decrease in diarrhea and other gastrointestinal diseases [17–20]. Boiling dominates the household water treatment landscape in resource-limited settings with data from a 67 country survey reporting more than 21 % of households preferring this method. Other practices included filtration, solar disinfection and the use of bleach or chlorine products [21]. In low-income communities, the improper handling and storage of water post treatment plays a large part in spreading water borne pathogens. In Peru for example, researchers looked at the fecal contamination of drinking water at different stages between the source and final consumption point. This work was carried out in a peri urban setting with chlorination at the main water outlets and household boiling. It was found that despite water at the source showing no microbiological contamination and boiling water before storage, almost a third of household samples from stored boiled water showed fecal contamination. This makes sense in the light of the fact that data collected on *E.coli* counts from the hands of users as well as from the cups residents used to pour water into before consumption showed high levels of bacterial contamination [22]. This decrease in quality of water between the collection at source and consumption has been seen in other community studies in countries like Brazil [23], Bolivia [24] and India [25] with factors like scooping water from containers with dirty hands, contamination during cleaning the storage vessel and the type of vessel used implicated as contributors to water contamination. Such studies highlight the importance of practices such as hand washing when collecting, handling and storing water. Obtaining enough quantities of clean water for hygiene and sanitation through methods like boiling or use of chlorine agents may prove expensive as well as time consuming [25]. The exposure of contaminated water to sunlight has consistently been shown to improve its quality and SODIS has so far primarily been used as a simple and cheap method to disinfect water for drinking. SODIS water treated in large bottles such as in WDCs can also be used to carry out hand and vessel washing in situations where the needs of drinking water are being met by other costlier methods. This can help to decrease the instances of recontamination of treated water in communities.

#### Emergency settings

In areas that have been struck by natural disasters or in refugee and disaster relief camps, the burden of providing necessary amounts of clean water falls on the local government and/or on aid organizations dealing with the crisis. It has been well established that provision of clean water for drinking is a critical factor in preventing morbidity and mortality in emergency settings [26]. However, it is worth noting that an equally important factor is an adequate quantity of clean water [27]. In light of the increased risk of susceptibility to diseases, the Sphere Project recommends that, following a disaster the provision of adequate quantities of water to the affected population takes precedence over access to high quality water until arrangements can be made to fulfil all minimum requirements [15].

According to the United Nations High Commissioner for Refugees (UNHCR), at the end of 2015 there were an estimated 21.3 million refugees worldwide [28]. A large percentage of these people live for varying amounts of time in over-crowded quarters in relief camps where basic water, sanitation and hygiene (WASH) practices can be severely compromised. As a result of such living conditions where the quantity of clean water is limited, a number of diarrheal disease causing pathogens including *E.coli* can spread infections mainly through fecally contaminated water [2, 26, 29]. Diarrheal diseases caused by contaminated water alone can be responsible for more than 40 % of deaths in such settings [26]. Activities such as maintaining personal hygiene, practicing good sanitation, managing medical and solid waste etc. are critical in preventing transmission of diseases caused by contamination through the improper storage, handling and transport of food and water and the sharing of cooking utensils [17, 26].

However in conflict areas, roads, waterways and other civil amenities may be disrupted thus making the transport of bottled water or of water in tankers to afflicted areas difficult, time consuming, unreliable and expensive. Following the recent civil unrest in Syria, refugee camps across the country were faced with acute water shortages resulting from compromised existing water supply systems. In the Yarmouk refugee camp near Damascus, people have been forced to depend on untreated groundwater obtained from wells. UNWRA medical staff reported that the severe water shortage coupled with crowded living conditions in the camp have led to increase in communicable diseases such as typhoid, scabies and diarrhea [30, 31]. The cholera outbreak in Haiti following the 2010 earthquake is another prime example of the importance of WASH conditions following a natural disaster. The measures to curb the rapid spread of the disease included transporting potable water to temporary shelters through tankers, chlorination of municipal water supplies and distribution of water treatment products [32, 33]. In disaster response as in the case of communities, recontamination of treated water following methods like boiling has been reported [34]. The importance of hygiene and sanitation can again be stressed in such circumstances - researchers investigating a Malawian refugee camp found that though the source water was relatively clean, contamination of water occurred by the hands of the women who collected the water in open rim buckets [35].

Studies examining intervention techniques for water treatment in refugee camps have previously reported success in curbing diarrheal diseases [35, 36]. SODIS in conventional containers is already a recommended method of treating water for affected populations in the wake of humanitarian crises or natural disasters [37]. Scaling up the volume of SODIS containers can prove useful in such settings where water treatment is usually done in bulk and not at a household level. Hence, SODIS in large volumes has the potential to cheaply and simply alleviate the problem related to water shortages in emergency situations – either as a means of water disinfection for consumption where costlier methods cannot be safely implemented or as a means of improving sanitation and hygiene to minimize the spread of fecal contamination.

### What are the challenges expected with SODIS in large volumes?

Based on an extensive literature review, we identified certain key concerns associated with the traditional SODIS method which may likely be carried over into any attempts to implement SODIS in large volumes. We discuss the following four challenges:

- Cold or Cloudy Weather [38]
- The Fear of Leaching in Plastic Bottles [39]
- Water Turbidity [38]
- Community Acceptance [40]

#### Cold or cloudy weather

Exposure to sunlight renders pathogens inactive by three possible mechanisms – optical inactivation, thermal inactivation and a synergistic action of both stresses. The synergistic effects that UV radiation and high temperatures have on bacteria have been well documented and this ‘combination therapy’ is seen to cause rapid drops in populations of viable bacteria when the temperature of the water being treated reaches 45 °C or more [41, 42].

Previous work concentrating on the dynamics of SODIS has looked at the effectiveness of small volume reactors under a variety of climatic conditions either through laboratory simulation of climatic conditions or field data. A mathematical solar radiation model constructed using field data in Haiti looked at the practicality of implementing SODIS all year round in the country. This work was carried out in January when the weather was colder and UV intensity less harsh when compared to Haiti’s normally tropical climate. 1.5 L PET bottles were used to evaluate the actual rate of bacterial inactivation and it was seen that instead of 5–6 h, a 2 day exposure was necessary to ensure 100 % success in microbial inactivation. The researchers saw no microbial inactivation on days when the incident solar intensity failed to meet the set threshold value, i.e. constant irradiation of 500 W/m^2^ for at least 3 h [43]. Given the synergistic activity of UV radiation and heat, the temperature that the water being treated reaches during exposure is critical. For instance, simulated sunlight experiments carried out on cysts of the protozoa Acanthamoeba polyphaga showed no appreciable reduction in cyst viability for water samples that reached a maximum of 40 °C over the course of 6 h. Subsequent work carried out by the same group reported a 3.6 log reduction in viable cysts at 50 °C after 6 h of treatment whereas a 3.3 log reduction was observed in only 4 h following exposure at 55 °C [44, 45].

When implementing SODIS, it is recommended that for best possible results the water to be treated be exposed to total solar irradiation of 500 W/m^2^ for about 5 h [6]. It is worth noting that several developing countries as well as most of the countries with the largest number of refugees and displaced people lie in or close to the global region perceived as being most favourable for SODIS [6, 28, 46]. However, emergency situations such as natural disasters can strike anywhere hence it may hence be necessary to evaluate the daily and seasonal variations in temperature and incident solar radiation on a particular site before the application of SODIS. In cloudy conditions, exposure times of up to 48 h are recommended for solar radiation levels to adequately disinfect water and under heavy rainfall, the use of SODIS is not recommended [6, 43]. Certain additives have shown success in enhancing SODIS’ effectiveness and reducing the overall time needed to disinfect water. A combination of SODIS and lime juice added to *E.coli* contaminated water in PET bottles showed a 5.6 log decrease in microbial cells within half an hour of solar exposure while SODIS alone showed a 1.5 log decrease [7]. Such findings highlight the potential usefulness of using additives to improve confidence in SODIS in situations where the radiation and temperature thresholds are not met. The study which determined the efficacy of WDCs as dependable SODIS vessels was conducted without any supplementation techniques, in the summer with the UV irradiance and temperatures being expectedly high. These conditions achieved a 5–6 log reduction in inoculated bacteria with maximum exposure duration of 6 h [16]. Future research is required on the applicability of WDCs and increased water volumes for SODIS treatment under varying climatic conditions and the accompanying temperature and solar intensity variations. Accordingly, the use of additives like limes or other supplementation techniques such as blackening the bottles’ surfaces can then be developed [7, 38].

#### The fear of leaching in plastic bottles

In the past, there have been public concerns about leaching of chemicals such as antimony and phthalates from plastic containers into the food or water they contain [39, 47]. As regards SODIS, Wegelin et al. filled PET bottles, exposed them to sunlight for up to 6 h and showed no harmful movement of photoproducts into the treated water under temperature and UV exposure encountered in field and laboratory tests [48].

Unlike small volume PET bottles, WDCs are generally made of polycarbonate (PC), a plastic whose main monomer is bisphenol-A (BPA), a chemical that is a known weakly estrogenic endocrine disruptor [49]. Studies have found that high temperatures and reuse can affect the rates of BPA migration from PC bottles into the water they hold [50, 51]. Brede et al. tracked the rate of release of BPA from PC baby bottles at elevated temperatures of up to 100 °C and under conditions of vigorous simulated use which included boiling, dishwashing and brushing at different temperatures. After an observation period of more than 20 h, a detectable amount of BPA was measured, though the levels did not exceed the then established limits for BPA exposure (0.01 mg BPA/kg body weight per day) [52]. This data taken together suggests that holding water in PC bottles at elevated temperatures, as would occur under SODIS treatment, would exacerbate the rate of BPA release from the plastic. It is worth noting however that these temperatures and time frames are much higher than those recorded in normal SODIS use. For instance, recent research on bacterial inactivation was carried out using 19 L PC large bottles as well as small volume PET bottles in the summer months in three countries - India, Bahrain and Spain. In each location, the bottles were exposed to 5–6 h of uninterrupted natural sunshine and temperatures recorded hourly. The water temperature never crossed 55 °C in any of the bottles under any of the experimental conditions [16].

Regulatory bodies have cleared the use of BPA in the food and beverage industry because recorded human exposure levels are considered too low to be able to cause any adverse health effects in any age group [53, 54]. Based on most recent evidences, the tolerable daily intake (TDI) for BPA from dietary exposure is 4 μg/kg body weight according to the European Food Safety Authority (EFSA) while the dietary intake in the highest exposure age groups is estimated to be 0.857 μg/kg body weight per day [54]. Separate BPA metabolism studies in rats and humans have shown that following oral exposure, BPA is efficiently expelled from the body and there is no evidence of any appreciable build-up of the chemical in the bloodstream or in the tissue. The human study examined serum BPA concentrations following dietary intake that was much higher than that reported for the general adult U.S. public. Nevertheless, no biologically active BPA was detected at the time of assessment [55, 56]. In the previously mentioned study by Brede et al., the most extreme case of leaching- with exposure to boiling water, multiple dishwasher cycles and brushing, measured BPA at 8.4 ± 4 μg/L (average from 12 bottles) [52]. In the light of the figures above on the TDI established by the EFSA, a person weighing 60 kg would have to consume around 28 l of the BPA tainted water in a single day to cross the tolerable limit (assuming no other dietary source of exposure).

A more recent study conducted using PET and PC bottles found that leaching of antimony and bromine into the water did occur and interestingly, the main contributor to the escape of these chemicals was not range of temperature or UV exposure, but number of times the container was reused. PC bottles reused up to 27 times showed antimony leaching into the water at concentrations of around 17 ng/L while bromine was measured at around 15 ng/L. These levels were deemed insufficient to cause health concerns in users, however it was also suggested that larger volume bottles may be subjected to harsh cleaning procedures involving high temperatures, cleaning chemicals and mechanical scrubbing and that these factors together could influence the rate of release of harmful additives into the water [57]. This is definitely an area that requires deeper study as it is likely that users of SODIS will reuse the bottles either due to economic reasons or because it would prove inconvenient to procure fresh bottles. Research in this area is of interest to the community as there is a general fear associated with placing plastic bottles under direct sunlight due to leaching of carcinogens that may occur.

#### Water turbidity

Turbidity is a factor that varies greatly in water sources from region to region. Highly turbid water reduces the efficiency of solar disinfection by decreasing the penetration of sunlight through the water thus protecting microbes from inactivation. The Swiss Federal Institute of Aquatic Science and Technology (EAWAG) recommend that raw water have a minimum turbidity of 30NTU before being subjected to SODIS treatment [6]. Studies in Kenya have shown that in water samples having turbidity more than 200NTU, around 1 % of the incident UV radiation was able to penetrate the water samples and disinfection occurred mainly by the thermal route when the temperature of the water rose above 55 °C [4].

Recent investigations on the performance of WDCs compared with PET bottles filled with turbid (100 NTU) water have shown that satisfactory inactivation occurred in both cases though the time of exposure needed for the WDCs was slightly higher [16]. This is encouraging and further work is needed that evaluates SODIS in WDCs singly as well as coupled with turbidity treatments as natural water turbidities can reach as high as 2000 NTU depending on factors such as rainfall [4].

There are several procedures recommended for removal of turbidity in water, including simple settling and filtration. Minerals like Alum (potassium sulphate) and the seeds of plants like *Moringa oleifera* have been used in water flocculation to reduce turbidity. Both these flocculants have been studied as pre-treatment options to clarify water prior to the use of methods such as chlorination or SODIS and have shown promising results. However, consideration must be given to the fact that addition of any pre-treatment step lengthens the overall time required for disinfection and hence choosing a viable pretreatment method depends very much on factors like local availability of the resource, manpower and cost [58, 59]. Other factors to consider are the changes in physical characteristics of the water treated; for instance unlike flocculation by alum, the use of *M.oleifera* seeds does not affect the pH and conductivity of the water and also generates an appreciably less amount of sludge [60]. However, a stumbling block in the uptake of this turbidity removal method may be the increase in organic content following treatment and the accompanying changes in odour or taste to the water, particularly after storage [61].

#### Community acceptance

Barriers to the successful dissemination of SODIS can include ignorance regarding the causes of diarrheal disease, reluctance to put in the time involved in addition to that of a normal workday or during seasonal increases in workloads, and an absence of water sources in close proximity to the user [40, 62]. In developing countries, 12 % of households without piped water rely on children below 15 years for collection of drinking water with girls twice as likely to carry this burden as boys. In Sub-Saharan Africa, more than 25 % of the population takes longer than 30 min for a single round trip of water collection. Globally, the percentages of households within 15 min of a source of water vary greatly with the most recent data reporting 97 % in Eastern Europe and 54 % in Sub-Saharan Africa. Rural households are less likely than urban ones to have a source of water nearby [63]. Field data is needed before recommendations can be made on the communities in which SODIS volume can be scaled up. However, given the above figures on primary water collectors and considering the dimensions and weight of filled WDCs, SODIS on large volumes may prove inconvenient unless the bottles are filled and exposed at or near to the site of use.

Cost of the larger bottles may also be a relevant factor to their uptake in communities and the prices of large plastic bottles vary around the world. In Uganda last year, the unit price of a PET bottle (2 L) was 0.50€ while that of a WDC (19 L) was 6.40€ [16]. Keeping in mind 15 L as the upper limit of water quantity needed per person per day and assuming no other means of disinfecting water, a single person would spend 4€ and need to fully fill and monitor 8 PET bottles to cross this benchmark. A single WDC on the other hand would provide 4 L more than the recommended volume within a single SODIS treatment time.

Community studies on WDCs are vital because in cases where compliance is high, strong links have been seen between using SODIS and drops in incidences of diarrhea; indeed there is a proven link between long term, faithful adherence to any water treatment method and associated health gains [64]. For instance, in an earlier 6 month study in India, the closely controlled and monitored group of children in the intervention arm (compliance rate 78 %) showed a 40 % decrease in all diarrheal episodes compared to the non-intervention group [13]. Two other high compliance studies in Cambodia and in South Africa with 95 % and 75 % user compliance, respectively, reported reduced incidences of diarrhea in the intervention groups and also stressed the importance of participant motivation and choosing specific methods of participant allotment into intervention and control groups [65, 66].

## Conclusions

Given the minimum requirements of 7.5-15 L of water/person/day, disinfecting large volumes by methods such as boiling or chlorination may not be cost or labour effective in developing countries or in humanitarian emergencies. Access to sufficient quantities of clean water for hygiene and sanitation can be as important as clean water for consumption when it comes to prevention of communicable diseases. SODIS in large volumes can potentially meet these needs.

We recommend that future research focus on addressing some of the concerns and gaps in research associated with scaling up of SODIS volumes. These include the potential for leaching of plastic additives into treated water, temperature profiles and climatic conditions under which WDCs perform well and the effectiveness of the method when coupled with pretreatment techniques. Moreover, close attention should be given to studying and developing dissemination procedures that guarantee the highest rates or compliance with SODIS. Social research is also needed to look at acceptance and perceived benefit of the SODIS at the community level, and the role it can play to improve the resilience of communities to social and economic shocks in conflict and post conflict settings.

## Abbreviations

ADI: Acceptable daily intake
BPA: Bisphenol A
CDC: Centers for Disease Control and Prevention
EAWAG: Swiss Federal Institute of Aquatic Science and Technology
EDI: Estimated dietary intake
EFSA: European Food Safety Authority
FDA: Food and Drug Administration
IDMC: International Displacement Monitoring Centre
PC: Polycarbonate
PET: Polyethylene terephthalate
SODIS: Solar water disinfection
TDI: Tolerable daily intake
UNHCR: United Nations High Commissioner for Refugees
UNWRA: United Nations Relief and Works Agency for Palestine Refugees in the Near East
WDC: Water dispenser container
WHO: World Health Organization
